# Supplementary material for: Ultrasound Evaluation of Internal Jugular Venous Insufficiency and Its Association with Cognitive Decline
Source: Diagnostics (Basel). 2025 Jun 4;15(11):1427. doi: 10.3390/diagnostics15111427 (PMC12155085; doi:10.3390/diagnostics15111427)
Supplement: Supplementary file 1 [file diagnostics-15-01427-s001.zip › diagnostics-3645190-supplementary.pdf]

Supplementary Figure S1. The flowchart of the patient recruitment

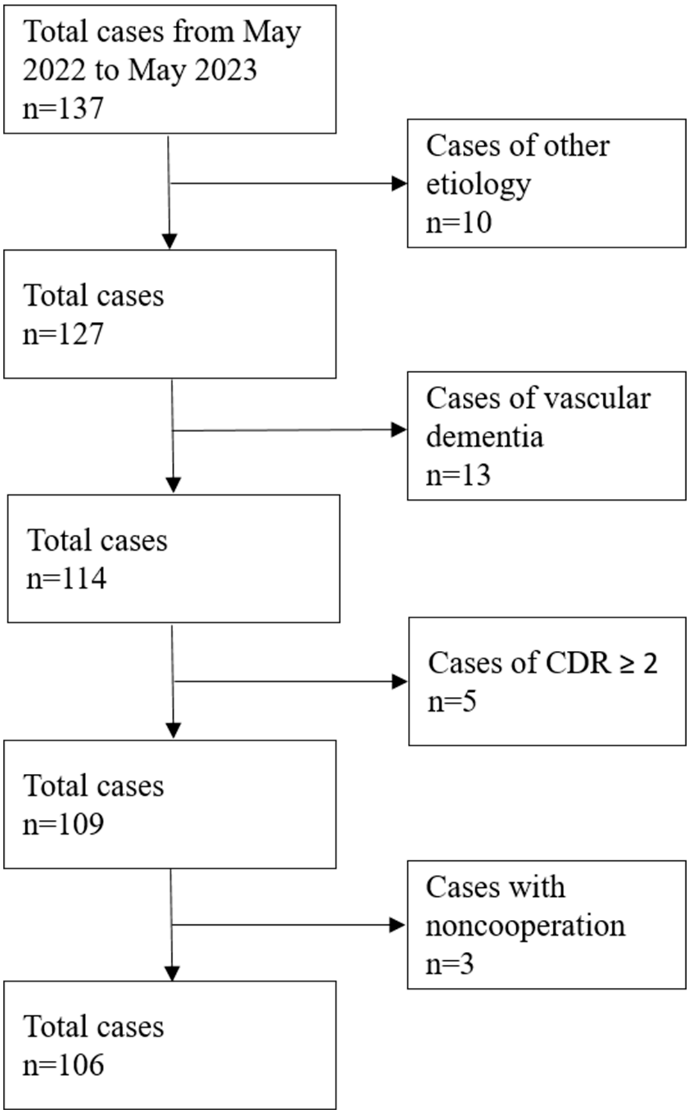

Supplementary Table S1. The Spearman correlation coefficient between the basic information of the research sample and its intelligence test. It showed a significant nega-tive correlation between age and neuropsychological test scores (MMSE: r=-0.564; MoCA: r=-0.701, P<0.0001) but significant positive correlation between years of edu-cation and neuropsychological test scores (MMSE: r=0.577; MoCA: r=0.689, P<0.0001)

	MMSE	MoCA	AGE	Edu	BMI
**MMSE**	1	0.750	-0.564	0.577	0.022
		<.0001	<.0001	<.0001	0.472
**MoCA**		1	-0.701	0.689	-0.058
			<.0001	<.0001	0.631

**Age**			1	-0.698<br><.0001	-0.085<br>0.560
**Edu**				1	0.041<br>0.736
**BMI**					1

Abbreviation: MMSE: Mini-Mental State Examination; MoCA: Montreal Cognitive Assessment; BMI: Body Mass Index

Supplementary Table S2. Ultrasound parameters of carotid duplex sonography and transcranial Doppler sonography

	**Cognitive impairment** (n=50)	**Normal cognition** (n=56)	*P*-value
Carotid duplex study			
LCCA (cm/sec)	41.5±10.5	44.5±11.1	0.2021
RCCA (cm/sec)	39.2±8.5	39.7±9.3	0.7516
LICA (cm/sec)	40.4±10.8	40.2±9.9	0.9269
RCCA (cm/sec)	39.6±8.6	38.3±10.2	0.2919
LVA (cm/sec)	23.8±8.0	23.3±6.1	0.1571
RVA (cm/sec)	23.8±7.2	22.7±6.4	0.2055
LCCA_PI	1.52±0.26	1.47±0.29	0.2354
RCCA_PI	1.42±0.27	1.43±0.19	0.7373
LICA_PI	1.10±0.22	1.02±0.24	0.2507
RICA_PI	1.08±0.19	1.02±0.20	0.1264
LVA_PI	1.26±0.25	1.21±0.28	0.1706
RVA_PI	1.28±0.26	1.24±0.32	0.5375
Transcranial Doppler study			
LMCA(P) (cm/sec)	57.8±18.6	64.6±23.0	0.0904
RMCA(P) (cm/sec)	57.9±14.0	63.1±21.5	0.0512
LMCA(D) (cm/sec)	59.5±19.6	59.1±18.5	0.9714
RMCA(D) (cm/sec)	57.1±11.6	63.1±21.5	0.2180
LMCA(P)_PI	1.01±0.18	0.93±0.19	0.0399*

RMCA(P)_PI	1.01±0.20	0.94±0.22	0.0737
LMCA(D)_PI	1.01±0.20	0.98±0.21	0.5360
RMCA(D)_PI	0.99±0.14	0.95±0.23	0.1631

Abbreviation: L: left; R: right; CCA: common carotid artery; ICA: internal carotid ar-tery; VA: vertebral artery; PI: pulsatility index; MCA: middle cerebral artery; P: proxi-mal segment; D: distal segment

*P<0.05
